# Prevalence and molecular characterization of canine parvovirus

**DOI:** 10.14202/vetworld.2021.603-606

**Published:** 2021-03-09

**Authors:** Parikshit Singh, Gurpreet Kaur, Mudit Chandra, P. N. Dwivedi

**Affiliations:** Department of Veterinary Microbiology, College of Veterinary Science, Guru Angad Dev Veterinary and Animal Sciences University, Ludhiana, Punjab, India

**Keywords:** canine parvovirus, nested polymerase chain reaction, polymerase chain reaction, sequence analysis, *VP2* gene

## Abstract

**Background and Aim:**

Canine parvovirus (CPV) belonging to family Parvoviridae causes hemorrhagic gastroenteritis in dogs and heavy mortality in young dogs. The virus has three structural (VP1, VP2 and VP3) and two non-structural proteins (NS1 and NS2), VP2 being highly immunogenic. This study aims to study molecular epidemiology of CPV by sequence analysis of *VP2* gene to determine the prevailing antigenic type(s) in the northern regions of India.

**Materials and Methods:**

A total of 118 rectal swabs collected from dogs exhibiting clinical signs of CPV infection were processed for the isolation of DNA and subjected to polymerase chain reaction (PCR) and nested PCR (NPCR). A total of 13 NPCR products selected randomly were subjected to sequence analysis of *VP2* gene.

**Results:**

The percent positivity of CPV was found 28% and 70% by PCR and NPCR, respectively. Dogs with vaccination history against CPV too were found positive with a percent positivity of 24.10%. Gene sequencing and phylogenetic analysis of *VP2* gene from these isolates revealed that most samples formed a clade with CPV-2a isolates.

**Conclusion:**

Sequence analysis and phylogenetic analysis of *VP2* gene in the studied regions of northern India revealed that CPV-2a was the most prevalent antigenic type.

## Introduction

Canine parvovirus (CPV) is a highly contagious virus and a common cause of acute, infectious gastrointestinal disease affecting young/unvaccinated dogs. It is among the smallest known viruses (~approx. 25 nm in diameter) belonging to the genus *Protoparvovirus* of the family *Parvoviridae* [1]. CPV is a non-enveloped icosahedral virion with a linear, non-segmented, negative sense, single-stranded DNA genome of 5323 bases which encodes for three structural proteins (VP1, VP2, and VP3) and two non-structural (NS) proteins (NS1 and NS2). VP2 is the major structural protein of capsid and its antigenic determinant. Earliest known CPV-positive sera were reported in Greece in 1974. CPV was first identified during mid-1978 as an emerging pathogen [2,3] and was referred to as CPV-2 to distinguish it from CPV-1 [4]. In India, CPV-2 was initially reported from Madras by Balu and Thangaraj [5] and was first isolated by Ramadass and Khader [6].

Some amino acid substitutions in *VP2* gene sequence can lead to mutations in CPV which is mainly responsible for resulting in different antigenic variants of the virus [7,8]. At present, there are four known antigenic types of CPV circulating throughout the world, namely, CPV-2, CPV-2a, CPV-2b, and CPV-2c. As the knowledge regarding current antigenic types of CPV in North India is still limited, more seroprevalence data and sequencing *VP2* gene for the detection of mutations would be of great help in the identification of the possible new emerging CPV strains.

Thus, the present study was designed to detect and identify the prevailing antigenic type(s) of CPV in different regions of North India.

## Materials and Methods

### Ethical approval

The Institutional Animal Ethics Committee permission was obtained through Memo No: GADVASU/2018/IAEC/47/19.

### Study period and location

The samples were collected from July 2019 to February 2020. The study was conducted at the Department of Veterinary Microbiology, COVS, Guru Angad Dev Veterinary and Animal Sciences University, Ludhiana.

### Collection of samples

A total of 118 rectal swabs were collected in 4 mL phosphate buffer saline (pH=7.2) from dogs exhibiting gastroenteritis/hemorrhagic enteritis with pyrexia, vomiting, dehydration, etc. Samples were collected from Friendicoes, New Delhi (n=35); Gurugram, Haryana (n=16); Chandigarh (n=6); Jammu (n=6); and the Small Animal Referral Veterinary Clinics, Guru Angad Dev Veterinary and Animal Sciences University (GADVASU), Ludhiana, Punjab (n=55). A rectal swab sample from a healthy dog too was collected and was used as negative control. A vaccine Nobivac DHPPi (Merck, Germany) was procured from a local drug store, Ludhiana, Punjab. The DNA was extracted from all the samples and the vaccine using the phenol-chloroform extraction method as described by Russell and Sambrook [9].

### Polymerase chain reaction (PCR) for the detection of CPV

The primers used in the PCR for the detection of CPV in rectal swabs were as per Mizak and Rzezutka [10]. The PCR reaction mixture was prepared by adding 15 μL of the template DNA, 5.0 μL of 10× PCR buffer (with 15 mM MgCl_2_), 1.0 μL of forward and reverse primer (20 pm/μL) each, 1.0 μL of dNTPs mix (10 mM each) (Takara Bio, USA), 0.2 μL Taq DNA polymerase (5 units/μL) (Qiagen, Germany), and the final reaction mixture volume was made up to 50 μL using nuclease-free water.

### Nested PCR (NPCR) for the detection of CPV

The primers for NPCR for the detection of CPV were as per Mizak and Rzezutka [10]. The NPCR reaction mixture was prepared by adding 5 μL of the PCR product (from above), 2.5 μL of 10× PCR buffer (with 15 mM MgCl_2_), 1.0 μL each of forward and reverse primer (20 pm/μL), 1.0 μL of dNTPs (10 mM each) (Takara Bio), 0.2 μL Taq DNA polymerase (5 units/μL) Qiagen), and the final volume was made up to 25 μL by adding nuclease-free water.

Both PCR and NPCR were set at thermocycling parameters with 35 cycles of denaturation at 94°C for 60 s, annealing at 55°C for 60 s, elongation at 72°C for 150 s, and a final elongation at 72°C for 10 min. Both PCR and NPCR products (10 μL each) were subjected to agarose gel electrophoresis using 1% agarose at the rate of 5 volts/cm with Gene Ruler ladder plus 100 bp (New England Biolabs, USA). The products on gel were visualized and documented using gel documentation system (Syngene, USA).

### *VP2* gene sequencing

A total of 13 NPCR products (Table-1) were purified using UltraClean PCR Clean-Up Kit (Mo Bio Labs., Inc., USA) and were submitted for sequencing (Eurofins Genomics, India Pvt. Ltd.). These sequences were analyzed and compared with the CPV sequences available from the GenBank using NCBI BLAST (http://blast.ncbi.nlm.nih.gov/blast.cgi) and Clustal Omega (www.ebi.ac.uk/tools/msa/clustalo/).

**Table-1:** History of CPV samples randomly selected for sequencing from various regions of North India.

Sample No.	Age (Months)	Sex	Breed	PCR	Nested PCR	Vaccination/booster status
D1	1.5	Female	Mongrel	+	+	−
D2	1.5	Male	Mongrel	−	+	−
P1	3	Female	Mongrel	+	+	−
P7	6	Male	Mongrel	+	+	−
P9	1	Female	Pomeranian	−	+	−
P10	1	Female	American Bully	−	+	−
P19	6	Female	Pomeranian	−	+	−
P47	7	Male	Mongrel	+	+	−
P48	3	Male	Labrador Retriever	+	+	Vaccinated and booster also given
P51	4	Female	Dachshund	+	+	−
C2	3.5	Female	Dobermann	+	+	Vaccinated
C6	4	Female	Cocker Spaniel	+	+	Vaccinated and booster also given
J1	4	Female	Mongrel	+	+	−

CPV=Canine parvovirus, PCR=Polymerase chain reaction

## Results

### PCR and NPCR

**Figure-1:**
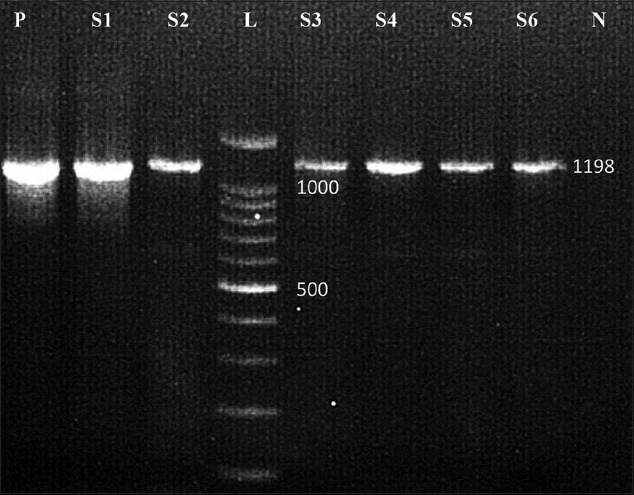
Polymerase chain reaction product visualization gel run. Lane L=100 bp plus ladder, P=Positive control, N=Negative control, S1-S6=CPV samples.

**Figure-2:**
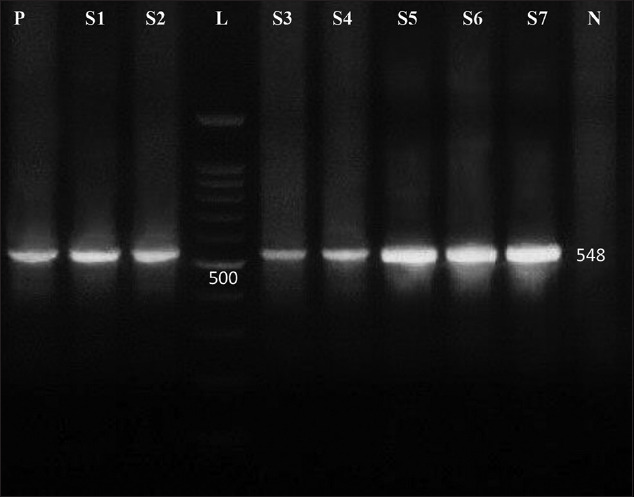
Nested polymerase chain reaction product visualization gel run. Lane L=100 bp plus ladder, P=Positive control, N=Negative control, S1-S7=CPV samples

Out of 118 CPV DNA samples subjected to both PCR and NPCR, a total of 33 samples were found to be positive for PCR with an incidence of 28% with a product yield of 1198 bp (Figure-1) and 83 samples were found to be positive for NPCR with an incidence of 70% with a product yield of 548 bp (Figure-2). Age-wise status of NPCR-positive samples indicated that 73.49% of samples belonged to dogs below 3.5 months of age. Sex-wise status of NPCR positive samples indicated that 60.24% samples belonged to males, while 39.76% samples belonged to females. Vaccination status of NPCR-positive samples indicated that 75.90% samples belonged to unvaccinated dogs, while 24.10% samples belonged to vaccinated dogs.

### Sequence analysis

**Figure-3:**
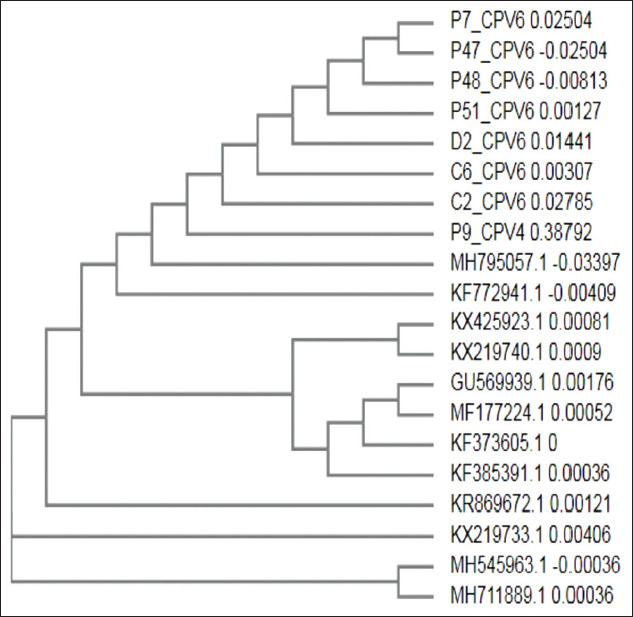
Phylogenetic analysis of canine parvovirus (CPV) samples with CPV 2a sequences from GenBank. Lane L=100 bp plus ladder, P=Positive control, N=Negative control, S1-S7=CPV samples.

**Figure-4:**
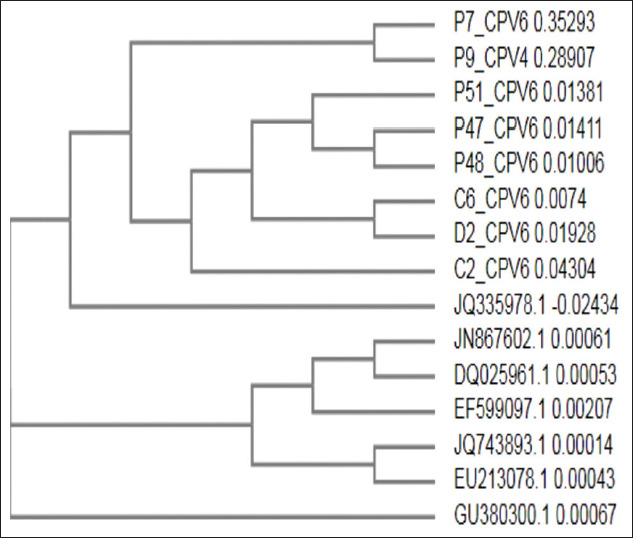
Phylogenetic analysis of canine parvovirus (CPV) samples with CPV 2b sequences from GenBank.

**Figure-5:**
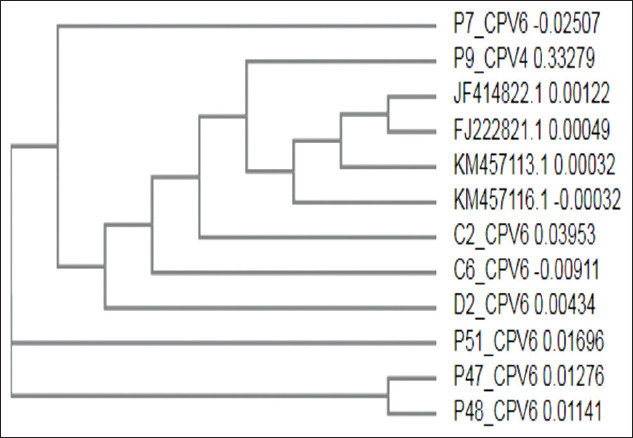
Phylogenetic analysis of canine parvovirus (CPV) samples with CPV 2c sequences from GenBank.

On the basis of NCBI BLAST analysis, it was found that the sequences had 99-100% homology with the existing CPV sequences. Phylogenetic analysis of subjected samples revealed that most of them formed a similar clade with CPV 2a isolates (Figure-3) and a separate clade with CPV-2b and CPV-2c sequences from GenBank (Figures-4 and 5).

## Discussion

Since the emergence of CPV in 1978, the virus had been known to continually evolve into many antigenic variants that can be found all over the world. This great antigenic variability of CPV due to its high mutation rates has raised a concern for the potential disease outbreak leading to negative impact on the health of primarily domestic dogs. Studying the ongoing status of geographical prevalence of particular antigenic type(s) is of key importance to keep a check on the mutating antigenic types to ensure the good health of dogs in future. In the present study, the prevalence of CPV antigenic types was studied in different regions of North India. The results of the present study aligned with the findings of many earlier researchers from India who did the sequence analysis of *VP2* gene of CPV to understand its prevailing antigenic types. Molecular characterization of field isolates of CPV and their sequence analysis of *VP2* gene revealed that CPV 2a was found to be the most prevailing antigenic type in the studied regions of North India, while NPCR was found to be a more sensitive molecular technique than conventional PCR for the detection of CPV. The present study also reinforced the established fact that among pedigreed dogs, breeds such as Labrador retriever, German shepherd, and Pomeranian were most affected, while dogs below 3.5 months of age were at greater risk and males were having more percent positivity. With the sequence analysis of *VP2* gene, the prevalence of CPV 2a has been commonly observed in many earlier studies [8,11-18]. Similarly, a few studies have also indicated the presence of both CPV 2a and CPV 2b [8,11] in the samples from dogs. Similarly, a new variant of CPV 2a and CPV-2b types circulating in the dog population of Brazil has been reported[19], and a new variant of CPV 2a was detected from Colombia [20].

## Conclusion

Sequence analysis of the *VP2* gene of CPV field isolates collected from the regions of Punjab, New Delhi, Haryana, Chandigarh, and Jammu revealed that CPV 2a was the prevalent antigenic type circulating in these regions. Further, continued studies are needed to keep record of new mutations of CPV antigenic types and their prevalence in different geographical regions for better understanding and preparation of new vaccine strains.

## Authors’ Contributions

GK designed, planned, and supervised the study. PS performed all the experiments and collected the data. PS, GK, MC, and PND analyzed the results. PS and GK wrote the manuscript. MC and PND edited and finalized the manuscript. All authors read and approved the final manuscript.
